# New decoding algorithms for Hidden Markov Models using distance measures on labellings

**DOI:** 10.1186/1471-2105-11-S1-S40

**Published:** 2010-01-18

**Authors:** Daniel G Brown, Jakub Truszkowski

**Affiliations:** 1 David R. Cheriton School of Computer Science University of Waterloo Waterloo ON N2L 3G1 Canada

## Abstract

**Background:**

Existing hidden Markov model decoding algorithms do not focus on approximately identifying the sequence feature boundaries.

**Results:**

We give a set of algorithms to compute the conditional probability of all labellings "near" a reference labelling *λ *for a sequence *y *for a variety of definitions of "near". In addition, we give optimization algorithms to find the best labelling for a sequence in the robust sense of having all of its feature boundaries nearly correct. Natural problems in this domain are *NP*-hard to optimize. For membrane proteins, our algorithms find the approximate topology of such proteins with comparable success to existing programs, while being substantially more accurate in estimating the positions of transmembrane helix boundaries.

**Conclusion:**

More robust HMM decoding may allow for better analysis of sequence features, in reasonable runtimes.

## Background

Decoding hidden Markov models (HMMs) continues to be a central problem in bioinformatics. Here, we move away from traditional decoding approaches, which seek to find a labelling or path optimizing a function of that single labelling or path, to a more robust method, where we seek a labelling of a sequence which has high probability of being close to the true labelling. As such, while the labelling we predict may not be correct, it has very high probability of being useful in a wide variety of standard HMM applications. One of our key observations is that since the primary use of HMMs is to divide sequences into features, we should focus on predicting feature boundaries nearly correctly. To that end, we introduce a distance measure for predictions where two labellings are "close" if they agree on the overall structure of the sequence and place feature boundaries at nearby sites. We seek the labelling for which the probability that the true labelling is "close" to it is highest, according to the probability distribution of the model. We also present a different weighted Hamming distance measure where we score each mismatch between predictions. We give efficient algorithms for computing the total probability of all HMM paths close to a given labelling. We also give an efficient local search optimization procedure for finding good labellings, and a global optimization procedure for a restricted version of the problem where we focus on paths through the model, not labellings. Computing the labelling with maximum nearby probability is *NP*-hard. Finally, we have implemented our methods, and show experimental results for predicting transmembrane protein topology. Our methods give results comparable to existing techniques such as Krogh's 1-best heuristic [1], implemented in the standard transmembrane protein topology predictor Phobius [2], at predicting the overall topology of membrane proteins. Moreover, they are more likely to get the boundaries of transmembrane helices in such proteins quite close to correct.

### HMM definitions

A hidden Markov Model (HMM) is a tuple *M *= (*A*, *E*, Σ, *x*_0_): *A *is the *m *× *m *transition matrix where *a*_*ij *_gives the probability of transition from state *i *to state *j*; *E *is an *m *× |Σ| emission probability matrix where *e*_*kσ*_is the probability of emitting symbol *σ *∈ Σ in state *k*, and *x*_0 _is the start state of the model. A *path *in an HMM is a sequence of states *x*_0_, *x*_1_, ..., *x*_*n*_; in step *i *of the execution of the model, we transition from *x*_*i*-1 _to a new state *x*_*i*_, and emit symbol *y*_*i *_according to the distribution of row *x*_*i *_of the matrix *E*. The HMM defines a probability measure over paths and sequences: the joint probability of sequence *y *= *y*_1_, ..., *y*_*n *_and path *x *= *x*_0_, *x*_1_, ..., *x*_*n *_is .

In a *labelled *HMM, we add a labelling function ℓ, which assigns to each state of the model (from 1 to *m*) a label, which typically corresponds to a sequence feature. For a path *x*, let *λ *= *λ*_0_, *λ*_1_, ..., *λ*_*n *_= ℓ(*x*_0_), ℓ(*x*_1_), ...,ℓ(*x*_*n*_) be its *labelling*. Many states may share a label, so many paths may also share a labelling.

In HMM decoding, we are interested in labellings, which assign a feature to each position of a sequence. Often, a labelling for a sequence will have many consecutive positions with the same label. Given a labelling *λ *= *λ*_0_, *f*_1_, *f*_1_, ..., *f*_1_, *f*_2_, *f*_2_, ..., *f*_2_, ..., *f*_*k*_, ..., *f*_*k*_, which consists of *λ*_0 _followed by a number of positions labelled *f*_1_, then a number of positions labelled *f*_2 _(which is different from *f*_1_), and so on, we define its *footprint *to be the sequence *f *= *f*_1_, ..., *f*_*k*_; this corresponds to the overall labelling of the sequence, but with the feature boundaries left entirely flexible. For a sequence of length *n*, its footprint may be much shorter than *n *in length, assuming it has long features.

For each label *z*, let *L*_*z *_be the set of states with label *z*; let *L *be the size of the largest *L*_*z*_.

### Distance measures for labellings

Here, we consider two different types of distance measures for labellings of the same sequence. The appropriate distance measure depends on the domain: our first type is more suited to short footprints corresponding to long features, while the second, based on Hamming distance, is more suited to long footprints.

Consider two different labellings *λ *and *λ' *for the same sequence *y*. We will define their *border shift distance d*(*λ*, *λ*') as a function of the boundaries of their features.

First, if *λ *and *λ' *have different footprints, they are incompatible; we will set their distance to ∞.

**Figure 1:**
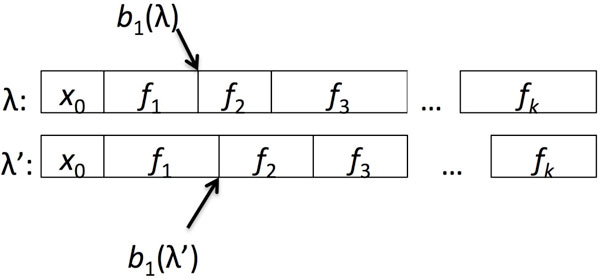
**The position of borders in labellings**. The *i*th border for a labelling *λ*, *b*_*i*_(*λ*), is the first position in *λ *with the label *f*_*i*+1_. The shift in the *i*th border, between two labellings *λ *and *λ' *that share the same footprint *f *is |*b*_*i*_(*λ*) - *b*_*i*_(*λ'*)|.

Otherwise, let *f *= *f*_1_, ..., *f*_*k *_be their common footprint, and let *b*_*i*_(*λ*) be the position in *λ *corresponding to the first emission from the interval labelled *f*_*i*+1_. The border shift distance between *λ *and *λ' *is then max_*i *= 1... *k*-1 _|*b*_*i*_(*λ*) - *b*_*i*_(*λ'*)|: the maximum boundary shift between the corresponding features in the two labellings. Figure 1 shows how we compute this distance.

We also define the *border shift sum distance*, which is infinite for unequal footprints, and  otherwise.

A quite different measure is based on counting mismatches between the labellings. In the *generalized Hamming distance*, we first assume we have a non-negative integer-valued distance matrix *H *that gives the distance between any two labels *i *and *j*. (In the easiest case, *H*_*ij *_= 0 if *i *= *j *and 1 otherwise; this gives the Hamming distance.) Then,  is the generalized Hamming distance between the two labellings.

For any metric *d *on labellings, we define the ball of radius *r *around a centre *λ*: *B*_*d*_(*λ*, *r*) = {*λ' *: *d*(*λ*, *λ'*) ≤ *r*}.

With this in mind, we state our goal. Given an HMM *M*, a sequence *y*, a distance metric *d*, and a distance tolerance *r*, we seek the labelling λ* such that Pr[*B*_*d*_(λ*, *r*)|*y*, *M*] is maximized. This labelling has, according to *M*'s probability measure, the highest probability of having the true labelling for the sequence be at most distance *r *away, according to our distance measure.

### Traditional algorithms

Algorithms typically used for decoding HMMs optimize quite different goals from ours. The most common are the Viterbi algorithm [3], which maximizes the probability of the state path used to generate a sequence, or the posterior algorithm, which maximizes the expectation of the number of positions in the sequence assigned to the correct state.

Both of these algorithms experience serious problems. The Viterbi path may itself have extremely tiny probability, and for long sequences, is highly unlikely to be correct overall; also, since this algorithm maximizes a global criterion, its prediction may be locally very sloppy. The posterior algorithm gives credible guesses for each position by itself, but does not identify intervals of the sequence as having common features; indeed, adjacent positions may be labelled with states that cannot co-exist in paths in the model with nonzero probability.

One might attempt to use generalized HMMs in this context, where the length distribution of a state is no longer geometric. However, this does nothing to allow the boundary between labels to be flexible, and may not help when multiple states share the same label.

One approach to hybridizing these methods is to compute the path of nonzero probability that maximizes the posterior decoding objective [4], or that maximizes the geometric mean of the probability of assigning correct states to positions [5]. These algorithms may improve results, but depend on the topology of the model being correct, and also may still give paths that are globally quite poor. There are variants of them that also work with labellings, not paths.

A tempting alternative might be to compute the most probable labelling of a sequence instead of the Viterbi path; this corresponds to our objective, but for balls of radius zero. This objective is *NP*-hard to optimize [6] in general, though Brejová *et al.*, in addition to proving the *NP*-hardness of the overall problem, do give an optimization algorithm for some special cases. Krogh [1] proposed the *1-best *algorithm, a heuristic to find a highly probable labelling, which is used in many applications, including transmembrane protein topology prediction [2]. However, even the most probable labelling may have very small conditional probability.

Several authors have also considered sets of paths rather than individual paths. For example, we could sample *k *paths from an HMM to predict alternatively-spliced exons [7,8]. In recent work, Brown and Golod [9] proposed finding the *k *most probable paths through an HMM, and applied this idea to transmembrane protein topology prediction. Our methods consider an exponential number of implicitly represented labellings, in contrast to these methods.

## Methods

### Computing the probability of a labelling and of a footprint

We begin with the simple problems of computing the probability of a labelling *λ *or of a footprint *f*. For both of these problems, our algorithm is a variant of the traditional forward algorithm for HMMs [3], which computes the probability that an HMM generates a sequence *y *in *O*(*nm*^2^) time.

To compute the probability of all paths compatible with the labelling, we follow the usual forward algorithm, but at position *i *in the sequence, we restrict to allowing only transitions in the model between states labelled *λ*_*i*-1 _and *λ*_*i*_; other transitions are given probability zero. This algorithm takes less time than the forward algorithm to run; if the largest number of edges between any two (possibly identical) labels *λ*_*i *_and *λ*_*j *_is *q*, then this algorithm takes *O*(*qn*) time, which is *O*(*L*^2^*n*).

**Figure 2:**
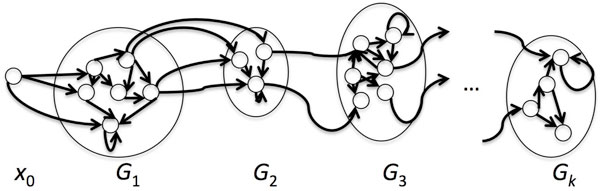
**Computing the probability of a footprint**. To compute the probability of a footprint *f *= *f*_1_, ..., *f*_*k*_, we create a group of states *G*_*i *_for each entry in *f*, corresponding to the states in *M *labelled *f*_*i*_. A path compatible with the footprint must first go from *x*_0 _to one of the states in *G*_1_, then eventually go to *G*_2_, and on to *G*_*k*_. Transitions are only allowed from *G*_*i *_to *G*_*i *_or *G*_*i*+1_.

To compute the probability of all paths compatible with the footprint *f *= *f*_1_, ..., *f*_*k*_, we follow the usual forward algorithm, but on a variant HMM. We will create *k *groups of states *G*_1_, ..., *G*_*k*_, each corresponding to a label set , for each position *f*_*i *_in the footprint: each state in  is represented in *G*_*i*_. States in the new model have exactly the same emission probabilities as in *M*, but we may only make transitions from states in *G*_*i *_to those in *G*_*i *_or in *G*_*i*+1_, with the same transition probabilities as in *M *. (To make a proper HMM, we can create a "dump" state for paths in *M *that do not respect the footprint we seek, or not bother.) See Figure 2.

We require that the initial transition is from *x*_0 _to a state in *G*_1_, and we compute only the sum of the forward probabilities ending in states in *G*_*k *_that have made all of the required label transitions in the footprint *f*. The new model has *O*(*Lk*) states and each state has degree at most 2*L*, so computing the probability of a footprint can be done using the forward algorithm in *O*(*L*^2^*kn*) time. Note that this could be quadratic in *n*, as *k*, the length of the footprint, is bounded above by *n*.

### Computing the probability of a ball of labellings

Computing the probability of *B*(*λ*, *r*), the ball of labellings around *λ *with radius *r *can be done efficiently for the border shift distance. As before, we produce a group of states *G*_*i *_for each position *f*_*i *_in the footprint *f *= *f*_1 _... *f*_*k *_of labelling *λ *. Each state in *G*_*i *_has nonzero transition probability only to states in *G*_*i *_or *G*_*i*+1_. However, if *b*_*i*_(*λ*) is the position of the *i*-th border in the labelling *λ*, then we allow states in *G*_*i *_to appear only in positions from *b*_*i*-1 _- *r *to *b*_*i *_+ *r *- 1. For each position *j*, we can identify the groups that can be active at that position in this way. Computing the ball probability is easily accomplished using the pattern for computing the probability of a labelling: we simply set the transition probabilities for transitions to a new label that are found outside of the correct interval to zero and forbid transitions within a label after its valid interval ends. We then run the forward algorithm on the updated HMM, which we call *M'*. *M*' has exactly the same states as for computing a footprint, meaning that the runtime to compute the probability of the ball *B*(*λ*, *r*) is *O*(*L*^2^*kn*). However, we can reduce this runtime. If *t*_*j *_is the number of groups of states *G*_*i *_that could be active at position *j*, then allowing the boundaries to shift by *r *makes *t*_*j *_go up by 1 at 2*r *positions. The asymptotic runtime is *q*∑*t*_*j*_, which is *O*(*qn *+ *qrk*). Again, since *q *≤ *L*^2^, this is *O*(*nL*^2 ^+ *rkL*^2^), which may be well less than the runtime of the forward algorithm on the original model *M*, depending on *r *and *k*.

For the border shift sum distance, we must remember the total amount by which we have already shifted the first *j *- 1 borders before we handle the next border. As such, we can imagine the same sort of HMM as for the maximum border shift, but with an additional parameter in each state to keep track of this total mismatch so far accumulated. The model winds up with *r *times as many states this way, but the set of active groups at each position, and the degree of each node in the new HMM, stay the same. The overall runtime is *r *times slower than for the maximum border shift distance, or *O*(*qnr *+ *qr*^2^*k*). We note also that the values of *r *chosen in practical applications are likely to be much larger for this distance measure than for the maximum border shift measure; as such, we expect that these algorithms are not likely to be as useful in practice.

Finally, for the generalized Hamming distance, with label distance matrix *H*, we can again hybridize the forward algorithm. Consider again our reference labelling *λ*, and create a new HMM with (*r *+ 1)*m *states, where each state's identifier is (*j*, *d*). The semantic meaning of being in state (*j*, *d*) at position *i *of the sequence is that the HMM *M *is in state *j *and we have accumulated *d *distance from *λ *in the first *i *positions of the sequence; we create such states for all *d *in the range from 0 to *r*. If there is a transition from *j *to *k *in the HMM *M*, and if *H*_*L*(λ{i=1}), *L*(*k*) _= *h*, then we draw an edge in our new HMM from (*j*, *d*) to (*k*, *d *+ *h*) for all *d *such that *d *+ *h *≤ *r*; if *d *+ *h *is more than *r*, then we do not allow the transition. The new HMM has (*r *+ 1)*m *states, each of which has the same degree as in *M*. We compute the total forward probabilities of all states (*j*, *d*) at position *n*, and this gives the probability of *B*(*λ*, *r*) for the generalized Hamming distance, in runtime *r *times that of the forward algorithm on the original sequence, or at most *O*(*m*^2^*nr*).

### Hardness results

We might hope that we can compute the labelling for which *B*(*λ*, *r*) is of highest probability for a given *r *and one of our distance measures. Unfortunately, this is *NP*-hard, because the problem of computing the maximum probability labelling is also *NP*-hard [6], even for a fixed HMM with only two labels.

Optimizing the ball with radius 0, *B*(*λ*, 0), is exactly this problem, so optimizing balls for general radius *r *is clearly *NP*-hard for either the border shift or border shift sum distances. Similarly, it is *NP*-hard for the generalized Hamming distance, since if we used radius *r *= 0 and the label distance matrix *H *with value 1 for mismatched labels and 0 for matched labels, we are again maximizing the probability of a labelling. We conjecture that this problem stays *NP*-hard when restricted to the pure Hamming distance on state paths, and not labels.

Furthermore, maximizing the probability of a ball of labellings under any natural distance remains *NP*-hard even if we restrict attention to a single footprint *f*. The original HMM used in the proof that maximizing the label probability is *NP*-hard has only two labels, so there are only 2*n *possible footprints for a sequence of length *n *in this HMM. As such, if there existed a polynomial time algorithm that finds a maximum probability ball for a given footprint *f*, we could solve the most probable labelling problem by setting *r *= 0 and running that algorithm for each of the *O*(*n*) footprints.

### Optimizing balls of states and paths: local search and special cases

Still, we can hope to find good balls of labellings using local search or global optimization in special cases. Here, we give a very efficient local search procedure to help optimize the probability of a ball of labellings using the border shift distance, and a global optimization procedure for computing the best centre for a ball of *paths*, not labellings, with the restriction that the path that forms the centre of the ball must divide the sequence up into long intervals, all using a single state.

#### Local search heuristic

The local search heuristic starts from a candidate labelling *λ *. At each iteration, it computes the probability of every ball centered at a labelling *λ' *matching *λ *in all borders but one, which is shifted by one position in either direction. We move to the *λ' *of highest ball probability and iterate until no improvement can be obtained by shifting a single border in the active *λ*. We can compute directly the ball probability for all 2*k *neighbours of *λ*; this gives runtime *O*(*k*^2^*rL*^2 ^+ *knL*^2^) per iteration.

We can improve this runtime by noticing that the active states at corresponding positions in *λ *and a neighbour *λ' *are identical for most sequence positions: if *λ' *consists of moving the border at position *j *in *λ *to position *j *+ 1, this only changes the active states at positions *j *- *r *and *j *+ *r*.

We can precompute the forward and backward probabilities of all states and positions for the ball of radius *r *around *λ*. For any HMM *M*, let *ϕ*_*i*_(*k*) be the probability that *M*, started at the initial state *x*_0_, emits *y*_1_, ..., *y*_*i *_and that *x*_*i *_= *k*, and let *β *_*i*_(*k*) be the probability that *M*, started at state *k*, emits *y*_*i*_, ..., *y*_*n*_. The probability that *M *emits *y *is the dot product of *ϕ*_*i *_and *β *_*i*+1 _for any *i*. We can compute the forward and backward vectors *ϕ*_*i *_and *β*_*i *_for all positions *i *in time proportional to the runtime of the forward algorithm. In particular, if we have computed Pr[*B*(*λ*, *r*)], we can also compute all of the forward and backward vectors at all positions of the sequence in the same asymptotic runtime.

Now, if we are considering moving the border at *j *to position *j *+ 1, we can keep the value of *ϕ*_*j*-*r*-1_, as the active states and probabilities are unchanged at the first *j *- *r *- 1 positions. From that point, we can use *ϕ*_*j*-*r*-1 _and run the forward algorithm in the slightly altered HMM with the new border for the next 2*r *+ 1 positions, until we compute the forward vector at position *j *+ *r*; call it . In the remaining positions, the probabilities do not change, so , and we can compute the ball probability for *λ' *by computing only *O*(*r*) columns in the forward algorithm. Each column might require as many as Θ(*rL*) active states in extreme cases, so the algorithm takes *O*(*krL*^2^) time to analyze each ball. (Note that *kL *is also an upper bound on the number of active states in any column, but this is likely a coarser upper bound, as *k *may typically grow with the sequence length, while *r *is a parameter of the optimization.) This method gives *O*(*nL*^2 ^+ *k*^2^*rL*^2^) runtime per iteration of the local search to compute the forward and backward vectors for *λ *and analyze all 2*k *neighbours *λ' *. In practice, this technique sped up our algorithms by a factor of ten for our experiments with transmembrane protein data shown later.

#### Global optimization for paths with long intervals

While the general problem of finding the most probable ball of labellings is *NP*-hard, the problem can be solved in polynomial time for a special case. Here, we look for the most probable ball of *state paths *rather than labellings (which corresponds to each state having its own label). Furthermore, we are only interested in finding the most probable among those balls whose centre paths have each state lasting at least 2*r *+ 1 positions. A path satisfying this requirement is called (2*r *+ 1)-*regular*. We call a path *π weakly *(2*r *+ 1)-*regular *if all its states except the final one last for at least 2*r *+ 1 positions, while the final state lasts at least *r *+ 1 positions. A *weakly *(2*r *+ 1)-*regular *ball is a ball centered at such a path. Our dynamic programming algorithm maintains variables *F *[*j*, *s*] corresponding to the probability of the most probable weakly (2*r *+ 1)-regular ball up to position *j *whose centre has state *s *at position *j*. The key idea behind the algorithm is that, by storing the probabilities of weakly (2*r *+ 1)-regular balls, we can ensure that the last state of each path in the ball is the same. This allows us to devise a dynamic programming procedure similar to the Viterbi algorithm.

An optimal weakly (2*r *+ 1)-regular ball up to position *j *can arise either by extending the final state of some centre path optimal to position *j *- 1 or by allowing a border to occur at position *j *- *r *because the last border must occur at least *r *positions previously. The recursive formula for calculating the probability of the new optimal ball is given by

where , the probability that the model emits the sequence *y*_*j*-2*r*-1 _to *y*_*j *_in only the states *s*' and *s*, and with a forced transition between those states in the interval, conditioned on starting the interval in state *s'*; we write  as a shorthand for *x*_*i*_, *x*_*i*+1_, ..., *x*_*j *_= *s*, ..., *s*.

In this formula, the first term computes the probability of the best weakly (2*r *+ 1)-regular ball ending at *s *at position *j *where there is a border at position *j *- *r*, and the second computes the probability of extending the previously optimal path in the same state.

Once we have computed the values of *F *[*j, s*] up to *j *= *n *- *r*, we multiply each of these values by a sequence of transition and emission probabilities corresponding to extending the state *s *up to position *n*. This ensures that our centres are (2*r *+ 1)-regular in the strong sense. Finally, we pick the largest probability obtained in this way and reconstruct the actual state path.

The runtime of the algorithm is determined by the time needed to calculate the *Q*(*s*, *s'*, *j*) values. We can calculate these sums in amortized constant time by reusing the values obtained for the previous value of *j*. This yields a runtime of *O*(*nm*^2^), as for the Viterbi algorithm.

## Results and Discussion

We have implemented our local search procedure and applied it to alpha-helical transmembrane protein topology prediction. In this problem, we are given a membrane protein sequence, and we are asked to label each amino acid as belonging to a cytoplasmic loop, a non-cytoplasmic loop, or a transmembrane helix. In any transmembrane protein topology, cytoplasmic and non-cytoplasmic loops alternate, separated by transmembrane helices, so the number of possible footprints is only linear in the length of the protein sequence.

Transmembrane protein topology predictors commonly use HMMs as a modelling tool. Examples of HMM-based approaches to this problem include Phobius [2], HMMTOP [10] and TMHMM [11]. Our methods are particularly appropriate for this problem domain for two reasons. First, researchers already have been using approximate correctness in helical boundary prediction. For example, the authors of Phobius [2] count a predicted topology as correct if its footprint is correct and the predicted location of each transmembrane segment overlaps with its true location in at least 5 positions, which bears some resemblance to our border-shift distance. Second, since the length of a transmembrane segment is bounded below by 15, while the average length of such a segment is 21, the number of possible footprints is low. By using our methods, we can be explicit about trying to approximate feature boundaries in transmembrane proteins.

### Data used

We used the larger of the two data sets used to train Phobius [2]. The data set contains 247 transmembrane proteins, whose average length is 385 residues. The average number of transmembrane helices per protein is 5. We also used the Phobius HMM for our experiments. The training procedure for the model was originally designed for use with the 1-best algorithm, which is used in Phobius. For details about how the model was trained, see the original paper [2].

We used two error measures to assess the accuracy of our algorithms. The first counts a prediction as correct if its footprint is correct and the predicted location of each transmembrane segment overlaps with its true location in at least 5 positions. As we noted above, the authors of Phobius used this measure to identify correct predictions, so we call it the "Phobius" measure. The second measure we used counts a prediction as correct if its border shift distance to the true labelling is at most 5. We call this the "*τ *= 5" measure.

We note that the *τ *= 5 measure is much more stringent than the Phobius measure. In our experiments, we discovered far lower success rates for decoding algorithms according to this measure than to the Phobius measure. That measure does not penalize predictions that move the borders of a transmembrane segment far away from its centre and also allows more error when the prediction considers a border to be too close to the centre.

### Initial labellings

We tested several different methods of choosing initial ball centres. For each of these methods, we then chose the ball of highest probability among those found by our local search procedure. In the first series of experiments, the initial ball centres were obtained by sampling from the conditional distribution of labellings given the protein sequence, using a standard algorithm [7,8].

The second initialization method that we tested was choosing labellings corresponding to the *K *most probable state paths through the HMM. The *K *most probable paths for each protein were obtained using the software by Brown and Golod [9].

Finally, to see if we could use the prediction of Phobius as a good starting point for local optimization, we performed local search on the single labelling obtained from the 1-best algorithm in Phobius, and from *K *- 1 sampled paths augmented by the single labelling from Phobius.

### Overall results

We compared the predictions of our methods with the predictions from Phobius. Results are shown in Table 1.

**Table 1:** Comparison of different algorithms.

Starting set	Local optimization	Phobius measure	*τ *= 5	runtime
10 samples, *r *= 6	Yes	162	92	132 m34 s
10 samples, *r *= 5	Yes	160	93	119 m53 s
Phobius	No	166	79	n/a
Phobius	Yes	166	94	8 m53 s
9 samples+Phobius	Yes	161	93	119 m33 s
10 best	Yes	141	75	116 m38 s

Comparison of accuracies for various algorithms tested. Shown are the numbers of correct predictions for various experimental conditions for two different correctness measures. We also show the total runtime of the local search procedure for all 247 proteins. For Phobius and 10 best, runtimes do not include the time needed to acquire initial labellings.

For this initial set of experiments, we used 10 samples from a variety of methods and optimized using our local search procedure.

Our methods are comparable to Phobius without local optimization for its measure of correctness (162/247 vs. 166/247). For our "*τ *= 5" measure, our optimized predictions from samples of the HMM space are significantly better (93/247 vs. 79/247). We used McNemar's test to verify that this result is significant at the 0.05 level for a 1-tail test.

In this experiment, our prototype implementation of our local optimization method required approximately 13 times as much time to compute predictions as did Phobius. However, we note that the parameters chosen (for *r*, for the number of iterations in the local search, and for the number of starting samples) were not done so as to reduce runtime, but to explore the effectiveness of our method.

### Robustness of results

The behaviour of the local search algorithm depends on several parameters: the radius *r *of the ball whose probability we seek to maximize, the number of starting points, and the maximum number of local search iterations. We ran our sampling procedure for a variety of parameter choices. Our method is robust to these parameter choices: most experimental setups differed in accuracy by at most 2 - 3 proteins according to the Phobius measure. For the "*τ *= 5" measure, accuracies differed in a range of about 7 - 8 proteins. Even for the latter measure, the only parameter that noticeably affected the results was the ball radius *r*. Increasing the number of starting points from 10 to 20 had almost no influence on the Phobius measure accuracy of the local search procedure with sampling (results not shown). True footprints of high probability were very likely to be sampled at least once with only 10 samples. On the other hand, if the true footprint had low probability, the local search procedure would likely have chosen a ball of higher probability instead, and more samples would not help.

Changing the maximum number of local search iterations also did not influence the results significantly. We tested our algorithms with 6, 20 and 80 local search iterations; this had no effect on the Phobius measure, while letting the algorithm come closer to convergence improved the "*τ *= 5" measure at at most 2 proteins. Changing the radius of the ball had little influence on accuracy according to the Phobius measure (at most 2 for all tested values of *r ≥ *2), but it did affect the performance according to the "*τ *= 5" measure. The number of correct predictions according to that measure varied from 84 (34%) to 93(37.6%) when we fixed the other parameters. Not surprisingly, the highest accuracy is achieved at *r *= 5. On the other hand, setting *r *= 6 yields the highest accuracy for the Phobius measure. Regardless of the radius used, the accuracies for the "*τ *= 5" measure were better than the accuracy obtained by the 1-best algorithm.

### Experimental conditions

Unless otherwise noted, we used *r *= 5 in an attempt to directly optimize the "*τ *= 5" measure. We also show the results for *r *= 6 for sampling since this radius value yielded the best accuracy according to the Phobius measure. For each algorithm, we used 10 starting points for the local search, except the one where the local search was carried out from the 1-best prediction from Phobius.

Substituting one of the random samples with the 1-best prediction does not noticeably change the accuracy of the local search algorithm. Local searches starting from the 1-best prediction rarely produce higher probability balls than local searches based on sampling. Running local search from the Phobius prediction alone substantially increases the "*τ *= 5" accuracy for those predictions while not giving a noticeable performance gain according to the Phobius measure.

Initializing local search from labellings corresponding to the 10 most probable paths yields substantially worse accuracies than the other methods. This may be because of a low diversity of footprints among these paths.

### Analysis of errors

We have also compared the sets of proteins predicted correctly by Phobius and by our sampling algorithm. The results are shown in Table 2. Among the proteins mispredicted by our local search algorithm, all but 2 have a footprint that is more probable according to the model than the true one. Of these two proteins, one is also mispredicted by Phobius. For all other mispredicted proteins, our algorithm predicts more probable, but incorrect, footprints. We have found that while correct solutions are often found for these proteins by some local search runs, they are discarded in favour of a wrong prediction having more probability mass around it. These results demonstrate that wrong predictions obtained by our algorithm are a result of the HMM itself rather than our decoding procedure. They also show the power of our local search algorithm combined with sampling.

**Table 2:** Error analysis. Shown are the numbers of correctly predicted proteins, the numbers of correctly predicted footprints, and the number of times each method predicted the most probable of the footprints found by the two methods and the true footprint.

Algorithm	correct footprint	Phobius measure	most probable footprint	*τ *= 5
Phobius	169	166	201	79
10 samples, *r *= 6	164	162	241	92

The efficiency of our local search procedure when combined with sampling is not surprising. Since we are trying to optimize for high probability balls, the probability of sampling a labelling somewhere within the most probable ball should be large. As a result, the distance between a sample and the centre of the most probable ball will often be small, which makes the local search procedure likely to achieve at least a good approximation of the most probable ball. Obviously, this strategy will perform worse if the most probable ball has low probability, but in such cases we are not likely to obtain a good prediction anyway from any decoding strategy.

## Conclusion

We have proposed a new decoding strategy for hidden Markov models: label a sequence in such a way that the true labelling of a sequence is likely to be close to the prediction. Unfortunately, this strategy is *NP*-hard, so we have proposed a highly efficient local search procedure to find good labellings using this approach in practice. We also provided a global search procedure for a special case of a variant of our procedure, where we focus on paths in the HMM and not labellings, and where we require that the path we return divides the sequence into long intervals all using the same state.

In our initial experiments, our local search procedure is comparable to existing algorithms at finding the overall approximate topology of a transmembrane protein, and for the ones that it does do a good job at approximating the topology, it is notably better at getting the boundaries of transmembrane helices almost exactly correct.

Our methods offer the possibility of a more robust method of HMM decoding, where instead of focusing on the correct answer, we can focus on finding annotations useful in subsequent applications. In future work, we shall apply these techniques to gene finding, to alignment, and to other applications of HMMs in bioinformatics and other domains. We will also consider training techniques to improve the accuracy of this decoding method.

## Competing interests

The authors declare that they have no competing interests.

## Authors' contributions

DB and JT jointly developed this decoding concept. JT developed most of the algorithmns, implemented them all, and performed the experimental work and wrote some of the manuscript. DB directed the project, wrote most of the manuscript and suggested the experiments we analyzed.
